# Effectiveness of 7.5 Percent Povidone Iodine in Comparison to 1 Percent Clotrimazole with Lignocaine in the Treatment of Otomycosis

**DOI:** 10.1155/2013/239730

**Published:** 2013-07-25

**Authors:** Ajay Philip, Regi Thomas, Anand Job, V. Rajan Sundaresan, Shalini Anandan, Rita Ruby Albert

**Affiliations:** ^1^Department of ENT Unit-1, Christian Medical College, Indian Subcontinent, Vellore 632004, India; ^2^Department of Microbiology, Christian Medical College, Indian Subcontinent, Vellore 632004, India

## Abstract

*Objectives.* Otomycosis is a common ENT disease frequenting the tropics. Its recurrent nature poses a great challenge to the treating physician. In spite of a number of antifungals in the market, the frequent nature of this disease warrants repeated use of these drugs, contributing to drug resistance and financial burden on the rural population. Our primary aims were to evaluate the effectiveness of povidone iodine in the treatment of otomycosis and to identify the most common fungal isolate in our population. *Study Design and Setting.* A single blinded prospective longitudinal study was done over a period of 12 months in a tertiary referral center. 34 patients in the age group 15–70 years clinically diagnosed with otomycosis were included in this study. These individuals were divided into two groups selected randomly. One arm received 7.5% povidone iodine otic drops and the other 1% Clotrimazole and lignocaine drops. Evaluation was based on resolution of symptoms and signs after treatment. *Result.* Both arms showed improvements which were comparable thus suggesting the role of povidone iodine in the management of otomycosis. *Conclusion.* Povidone iodine is an effective antifungal in the treatment of otomycosis.

## 1. Introduction

Fungal otitis externa (otomycosis) is a common disease throughout the world, with frequency varying in different geographic zones. It is prevalent in the tropics [1] and is sometimes associated with complications, involving the middle ear [2]. It has been an entity which has perplexed many otologists because of its recurrent nature.

Since its description about 100 years ago by Andral and Cavarret in 1843 and by Mayer in 1844 [3], many attempts have been made in the diagnosis and treatment of this condition. The disease is seen worldwide and is estimated to constitute approximately 5–25% of the total cases of otitis externa [4].

In a routine otolaryngology clinic, its prevalence ranges from about 9 percent to about 30.4 percent in individuals presenting with otitis externa [5]. It was believed that fungi were secondary invaders in external canal infections accounting for one third of the external otitis cases, and the remaining being attributed to gram negative-Bacilli (Syverton et al. [6]) [3].

Traditionally, the treatment of otomycosis revolved around good personal hygiene and avoidance of self-cleaning. But the management varied as time progressed which ranged from copious lavage to insertion of metacresylacetate wick, boric acid, and sulphathiazole ointment and instillation of topical ketoconazole, cresylate otic drops, and aluminum acetic drops [5]. The use of metacresylacetate, however, produced dermatitis and was subsequently discontinued. In recalcitrant cases, treatment with 75 rads of X-rays [7] has also been attempted. The medical treatment has abundant literature describing the use of various classes of drugs, mainly antifungals, in the topical treatment of otomycosis [8].

However there has never been a study which evaluated the role of iodine as an antifungal in otomycosis. It is a well-known fact that iodine is a routinely used antiseptic solution in surgical wards, with no documented resistance thus far. This study was undertaken to evaluate the antifungal [9] property of povidone iodine and to provide a cheaper alternative in the treatment of otomycosis.

## 2. Materials and Methods

The purpose of the study was to evaluate if povidone iodine was effective in treating clinically diagnosed fungal otitis externa. A single blinded prospective randomized case control study was done in our institute over a period of 12 months. The institutional research board clearance was obtained prior to the commencement of the study, and a detailed informed consent was taken from the subjects. 

All patients with clinically diagnosed otomycosis within the age range of 15–70 years were included in the study. They were subdivided into age groups 15–30, 31–50, and 51–70. Individuals with chronic suppurative otitis media, postoperative mastoidectomy cavities, malignant otitis externa, uncontrolled diabetes, hearing aids, and those undergoing chemotherapy or postchemotherapy status were excluded.

### 2.1. Target Sample Size and Rationale

A sample size of 270 was calculated. Sample size calculations were based on the fact that the primary outcome for this study was improvement in symptoms and cure. It was estimated that 90% in the Clotrimazole group will improve compared to 75% in the povidone iodine group. To find a 15% difference in the outcome, assuming the power to be 80% and level of significance to be 5%, sample size was calculated as 135 in each arm (total sample size = 270). For two groups with percentages (cure rate), Clotrimazole (*p*1) = 90% and proposed cure rate of povidone iodine is (*p*2) = 75%, sample size is calculated by sample size (*n*) = (*Z*@+*Z*1-*b*)22*PQ*/*d*2,  where *P* = (*p*1 + *p*2)/2 = (90 + 75)/2, *Z*@ = type  1  error = 1.96, *Z*1-*b* = type  2  error = 1.28, *d* = difference  in  outcome = 15, and *Q* = 100% − *P* = 100 − 82.5 = 17.5.

Therefore *n* = 10.4 × 2 × 82 × 17.5/225 = 134.7 = 135 patients in each arm. Since this is the first study using povidone iodine in clinically diagnosed cases of otomycosis, we planned to do a pilot study.

### 2.2. Method of Allocation Concealment

Each of the drugs was concealed in containers covered externally. The drugs were given in an open method and were dispensed by a trained staff nurse. The principal investigator, staff nurse, and the patient were unaware of the drug being administered. 

The study was done in the ENT department at our tertiary hospital The Christian Medical College and Hospital, Vellore. All patients who presented to the outpatient department with symptoms of itching of the ear, ear ache, ear discharge, blocked sensation in the ear, tinnitus, or hard of hearing were evaluated. The ear was inspected with an otoscope, and a clinical diagnosis of otomycosis was made, based on the history and examination findings of matted hyphae, spores, or curdy precipitate in the external auditory canal. The patients who fulfilled the above criteria were referred to the principal investigator.

The principal investigator re-examined these patients, visualized the ear under an operating microscope (Karl Zeiss) with a 250 mm objective lens, and noted the findings.

**Figure 1:**
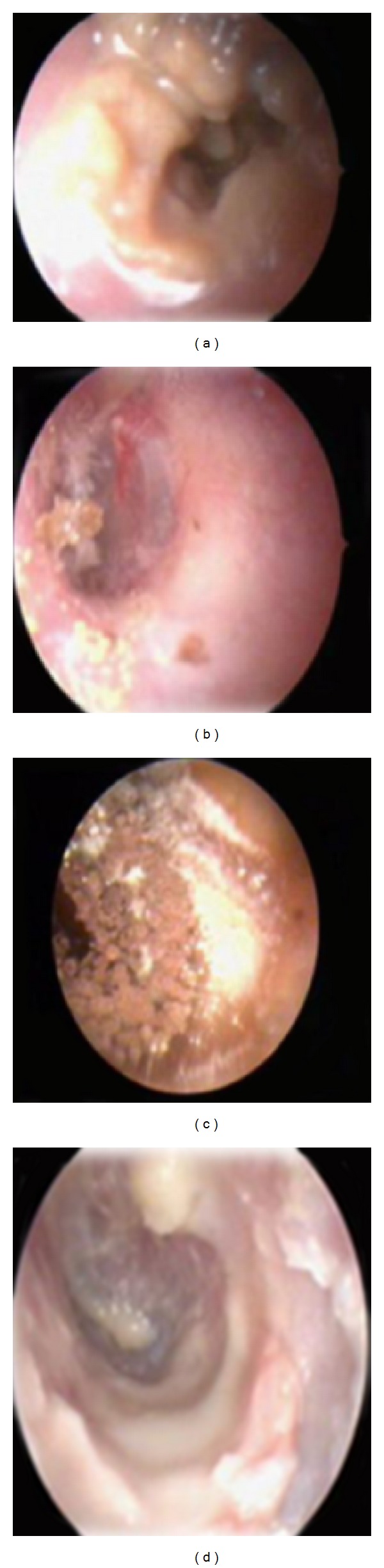
Otomycotic debris and spores in relation to the ear canal.

Presence of otomycotic debris (Figure 1) in the ear canal was classified as, lying in relation to the isthmus, bony canal or in the cartilaginous canal. 

Erythema of the cartilaginous canal and bony canal were looked for, and the status of the tympanic membrane was noted, whether congested or perforated. Otomycotic debris was suctioned out, and part of the debris was sent for a fungal smear in a sterile test tube containing saline.

Fungal debris were teased onto a slide and stained with gram stain. The ear swabs for this study were inoculated onto blood agar (BA) and Sabouraud's dextrose agar (SAB) with antibiotics and thioglycolate broth. BA was incubated at 37°C in CO_2_ atmosphere and SAB & thioglycolate broth at 37°C for 18 hours. If there was a suspicion of fungal growth at the point of inoculation, the plates were incubated for a further 24 to 48 hours till sporulation occurred in order to facilitate identification. Fungal growth was identified by doing Lacto Phenol Cotton Blue (LPCB) preparation. After thorough toileting of the ear, the patient was sent to the ENT treatment room for administration of the drug by the nurse in charge. All drugs were dispensed serially according to the randomization order generated by a computer.

 In the scenario where bilateral ears were affected, the more severely affected ear was taken as the test ear. The instillation was done with the patient lying on his/her non- affected side or the side with fewer symptoms with the affected ear facing upwards. The drops were instilled, and the patient was asked to maintain that posture for about 10 minutes. The patients were instructed to instill 3 drops of the drug into the affected ear once a day after thorough cleansing of hands, to be repeated daily for 13 consecutive days. At the end of 2 weeks, the patient was reviewed in the OPD and questioned regarding relief, persistence, or worsening of any symptoms and appearance of any new symptoms.

The ear was re-examined under the microscope; the findings noted and a repeat culture were taken from the ear. In the absence of debris, a smear from the canal wall was taken. A favorable outcome was obtained if the patient had no symptoms, his/her external auditory canal was free of debris, and the posttreatment smear showed normal flora or no growth.

## 3. Results

**Figure 2:**
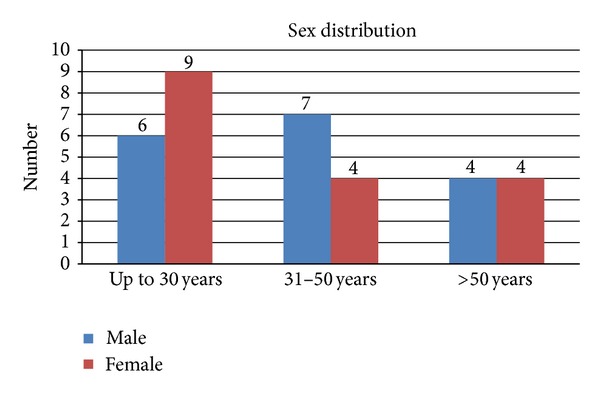
Sex distribution and disease.

The number of males and females in the three age groups was compared. A female predominance was noted in the first age group, a male predominance in the second age group, and equal predominance of both the genders in the third age group (Figure 2). The “*P*” value was 0.524.

**Figure 3:**
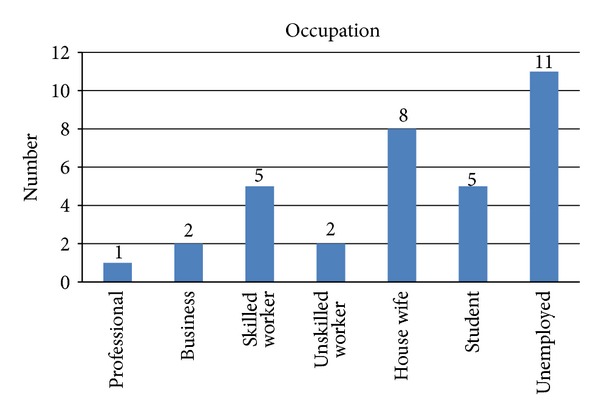
Relation of occupation with disease.

Unemployed individuals accounted for the maximum number of cases, with housewives comprising the second most affected group (Figure 3).

74% of the individuals had their left ear involved, with the remaining 26% having right ear disease. Pruritus was the most common symptom seen in 76.5% of the individuals followed by ear discharge, ear fullness, otalgia, tinnitus, and deafness (Table 1).

Tympanic membrane was congested in 67.6% of the individuals, followed by bony canal wall erythema, cartilaginous canal wall erythema, tragal tenderness, cartilaginous canal wall edema, ear discharge in the canal, and bony canal wall edema. Out of 34 individuals, 24 had history of self-cleaning and 24 had no wax in the ears. This probably explains the antifungal properties in wax (Table 2).

**Figure 4:**
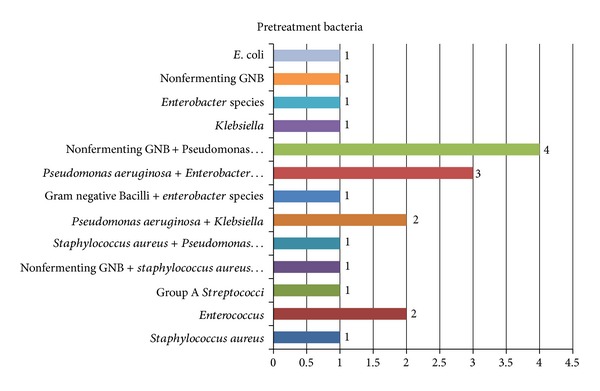
Pretreatment bacterial isolates.

In pretreatment ear swabs, (nonfermenting Gram negative Bacilli  +  *Pseudomonas aeruginosa*) accounted for 11.8% of the cases while (*Pseudomonas aeruginosa*  +  *Enterobacter*) species and *Staphylococcus aureus* formed 8.8% of the cases (Figure 4).

**Figure 5:**
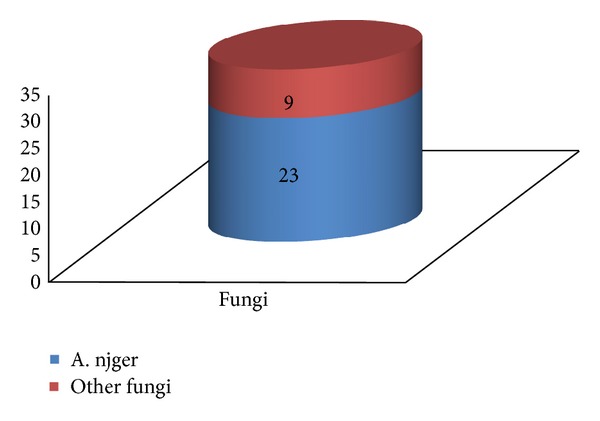
Moat commonly isolated fungi.

Among fungi, *Aspergillus niger* and *Aspergillus flavus* were most commonly isolated (23.5%) with *Aspergillus niger* with yeast constituting 11.7% of the cases. *Aspergillus niger* was the most common fungi, forming 60.86% of the isolates either in association with other fungi or in isolation and hence was the most commonly identified species in our institution (Figure 5).

After treatment with povidone iodine, nine of the smears grew *Pseudomonas aeruginosa*, which was the most common bacterial isolate followed by *Enterobacter*.

Following treatment with Clotrimazole and lignocaine, 3 had grown bacteria, with *Pseudomonas aeruginosa* forming the most common isolate followed by nonfermenting Gram negative Bacilli. Considering fungal infections, in the posttreatment group, 23 of the patients had no fungal growth, 8 were lost to follow up, and 3 had residual fungal infection out of which 2 belonged to the povidone iodine group and 1 in the Clotrimazole group.

In the symptom profile, the Clotrimazole group showed a resolution of 83.3%, 91.7%, 83.3%, 91.7%, and 91.7% in pruritus, ear discharge, fullness, tinnitus, and deafness, respectively. However there was a 100% resolution of otalgia. In the povidone iodine group, there was complete resolution of deafness and tinnitus, whereas pruritus, ear discharge, and ear fullness had 93.3% resolution. Otalgia resolved only in 86.7% of the cases.

Considering tragal tenderness, 100% had relief using both ototopical preparations. There was no statistical significance between the 2 groups (*P* value > 0.05); hence, the groups were only comparable. Povidone iodine had shown 100% resolution of both the cartilaginous wall erythema and the edema, whereas the Clotrimazole group showed favorable responses of 83.3% and 91.7%, respectively. Bony canal erythema had resolved in 91.7% and 93.3% of the cases, respectively. Clotrimazole showed a 100% resolution in the bony wall edema, ear discharge, and tympanic membrane congestion whereas povidone iodine showed a resolution of 93%, 100%, and 86.7%, respectively. It was not statistically significant (*P* > 0.05) and hence the two groups were comparable (Tables 3 and 4).

In view of the above findings, it can be stated that both drugs are equally efficacious in resolving the above symptoms and signs. But this could not be established as the statistical value was insignificant. Hence we recommend a study with a larger sample size.

## 4. Discussion

Otomycosis, a common condition encountered in any ENT practice, can often be a challenging and frustrating entity for both patients and treating otolaryngologists. Though complications are infrequent and rarely life threatening, it often recurs and may require prolonged treatment and followup. The aim of treatment is not only to cure the infection but also to alleviate the symptoms and signs caused by this condition. The high recurrence is attributed to the persistence of spores. Chronic suppurative otitis media, postoperative mastoidectomy cavities, and immunocompromised individuals are well-documented predisposing factors for this condition. Hence in our study, we have excluded the above conditions and tried to analyze if there are other predisposing factors of otomycosis. For complete disease clearance, management should address the underlying factors.

The basic principles of management of fungal otitis externa include having effective aural toilet, identifying the causative organism, and eliminating it using the appropriate antifungal agent. Though systemic antifungals have been attempted in otomycosis, topical preparations are commonly used as these fungi cause superficial infections only. Frequent relapses of otomycosis have been encountered due to the persistence of spores. Studies have shown that subepithelial spores persist in spite of using topical antifungal eardrops and hence stressed the importance of longer duration of treatment and followup [7]. However in our study, on examination after 2 weeks of treatment, no spores were encountered. Hence we emphasize meticulous aural toileting, especially in the region of the isthmus and the anterior recess. A number of ototopical antifungal treatments have been used in the past; these include application of antiseptics such as gentian violet, boric acid, cresylate, and aluminium acetate (Burrows Solution). However, these drugs fell out of favor in view of their ototoxicity, especially when the condition was associated with a tympanic membrane perforation.

Clotrimazole is a common antifungal of the azole group used in the treatment of otomycosis. It is used commonly in combination with either topical antibiotics or steroid preparations. The drug was found to be effective in most other studies, achieving a cure rate of 95% [8].

We planned to look at the efficacy of 7.5% povidone iodine solution in the management of otomycosis. Clotrimazole 1% with 2% lignocaine in propyl glycerol base was used as the control drug. Our primary aim was to find an alternative to Clotrimazole in the treatment of otomycosis.

We selected povidone iodine as it was easily available and has been proven to be effective in chronic suppurative otitis media which is one of the predisposing factors of otomycosis [10]. It is chemically stable, inexpensive, and resistance in bacteria and fungi is yet to be reported. Excessive and indiscrete use of any topical antibiotic and antimicrobials may lead to the emergence of resistant organisms. Povidone iodine overcomes this problem as there are no studies to date showing development of resistance, which is an increasing cause of concern in this era. In developing countries like India and the third world countries, where a cheaper and effective form of medication without ototoxicity is a requisite, povidone iodine forms a better choice.

We removed confounding factors such as immunocompromised individuals and patients with hearing aids and tympanic membrane perforations as they would require longer duration of therapy, and 2 weeks of treatment may not have sufficed.

Considering the demographic details of otomycosis, a retrospective study done in Shanghai in 2010 [11] concluded that females were more affected with a female : male ratio of 2 : 1. A similar result was seen in a study in northern Iraq [12]. However in our study, we found an equal preponderance in both sexes. Yet another retrospective study by Gutiérrez et al. [13] and a study in Nigeria showed a male predominance in this condition. Hence we conclude that there is no sex preponderance to this disease though a larger sample size may be required for the same.

Very few studies have described sex and age distributions, and the affected individuals mainly belonged to the 30–40-year age groups [2, 14]. In our study, a larger number of individuals belonged to the age group 15–30 with a female preponderance. This was however not statistically significant. Unemployed individuals were seen more affected with the disease followed by housewives. The various factors taken in comparison of the drugs, such as the patient's symptoms and signs, were compared pretreatment and posttreatment and tabulated.

 This is the first study in the literature where we have graded signs and noted the resolution of signs after treatment, and hence we propose grading of otomycosis based on the signs. The signs and symptoms in this study to each drug were later Chi squared and found to be insignificant (*P* value > 0.05), and so the groups were comparable.

Majority of our patients presented with pruritus followed by otorrhoea, ear fullness, otalgia, tinnitus, and deafness when compared with other studies such as that of Ho et al. [5] in which otalgia was the major symptom followed by otorrhoea and hearing loss.

Pruritus would have instigated the individual to self-clean his ear, traumatizing the epithelium, thereby leading to maceration and causing introduction of fungal and bacterial organisms, thus leading to infection. In our study, repeated self-cleaning seemed to have a close relation to the absence of wax. Majority of the patients had their left ear affected. This probably would have been related to the handedness of the general population which comprises of mostly right handed individuals. Use of the nondominant hand would have incurred more epithelial damage and hence infection. Further studies can be done including the handedness of the individual and ear side affected.

Looking at the microbial floral growth, studies have shown that mixed infections are quite rare as the fungi generally tend to inhibit bacterial flora [15]. In our study of clinically diagnosed cases of otomycosis, infection due to mixed flora topped the list, and we propose it may be due to formation of biofilms which are known to be quite resistant to the topical agents commonly used. This may also explain the recurrent nature of otomycosis. Further research will be necessary to confirm this. The commensals residing in the ear of a normal individual are *Staphylococcus epidermidis*, *Corynebacterium* spp, *Bacillus* spp, Gram positive cocci (*Staphylococcus aureus*, *Streptococcus* spp, nonpathogenic *micrococci*), Gram negative Bacilli (*Pseudomonas aeruginosa*, *Escherichia coli*, Haemophilus influenza, and Moraxella catarrhalis), and mycelial fungi of the *Aspergillus* genus or yeast-like fungi, particularly *Candida* spp.

Our pretreatment smears had a predominance of nonfermenting Gram negative Bacilli and *Pseudomonas aeruginosa*, and 75% of this group had association with fungus. Our studies tallied with most of the others in isolating *Aspergillus niger* as the most common species. When a person developed symptoms, it often denoted a disharmony between the pathogens and the onset of infection. Presence of Enterococcus and *Enterobacter* in our study suggests a faeco-aural transmission of the bacteria.

Though both Clotrimazole and povidone iodine groups had improvement in the posttreatment symptoms and signs, both had a residual fungal disease of 16.6 percent and hence povidone iodine had a cure rate equivalent to Clotrimazole. Povidone iodine has been shown to kill most of the bacteria in a biofilm, if not all as proved in in vitro studies. However it is less effective than hydrogen peroxide in biofilms. Hydrogen peroxide is irritant to the skin, and its application to an already erythematous ear canal may cause the patient more discomfort [16]. It causes effervescence and must be used only under supervision. Alcohol rapidly destroys biofilms of *Staphylococcus epidermidis*. *Candida* has been known to form biofilms especially in dwelling catheters, and studies are now suggesting biofilm formation of *Aspergillus* species with bacteria [17–19]. There has been no literature showing biofilms as a cause for recurrent external auditory canal infection; hence we strongly suggest that recurrence and persistence of disease are not just as a result of spores but probably also due to biofilm formation.

Oral antibiotics are indicated when coexisting bacterial infection results in incomplete resolution of the canal infection or when cellulitis of the external auditory canal sets in. Unlike other studies which compared only the symptoms and the culture, we categorized the signs as confined to various parts of the ear canal and the tympanic membrane and concluded that most of the patients had tympanic membrane congestion followed by bony and cartilaginous wall erythema. Fungal debris was most commonly noted in the bony cartilaginous isthmus, suggesting a defective epithelial migration and failure of normal lateral excursion in removal of the offending organism, thus forming a nidus for fungi to thrive on. A once a day application of the ear drops was done based on the study done earlier by Nong et al., which showed a cure rate of 95% [8]. As povidone iodine results were comparable with the Clotrimazole group, povidone iodine may also be applied once a day hence increasing the patient compliance. None of our patients developed any symptoms and signs of allergic contact dermatitis.

## 5. Conclusion

The result of this study supports the use of povidone iodine in the treatment of otomycosis, thus avoiding emergence of resistant organisms. Further studies with a larger sample size are necessary to provide a statistically significant value to gauge the more effective drug. This study has opened a window in the application of povidone iodine in clinically diagnosed cases of otomycosis in humans in addition to the management of chronic suppurative otitis media.

## Figures and Tables

**Table 1:** Symptoms—pretreatment.

Variables	Yes	No
Pruritus		
Yes	26	76.5
No	8	23.5
Ear discharge		
Yes	19	55.9
No	15	44.1
Otalgia		
Yes	18	52.9
No	16	47.1
Tinnitus		
Yes	10	29.4
No	24	70.6
Deafness		
Yes	7	20.6
No	27	79.4

**Table 2:** Self-cleaning and presence of wax.

Variables	Yes	No
Self-cleaning		
Yes	24	70.6
No	10	29.4
Wax		
Yes	10	29.4
No	24	70.6

**Table 3:** Symptom resolution with povidone iodine and clotrimazole.

Variables	Drug				*P* value
	Clotrimazole + lignocaine		Povidone iodine		
	*n*	%	*n*	%	
Pruritus					
Yes	2	16.7	1	6.7	0.569
No	10	83.3	14	93.3	
Ear discharge					
Yes	1	8.3	1	6.7	1.000
No	11	91.7	14	93.3	
Ear fullness					
Yes	2	16.7	1	6.7	0.569
No	10	83.3	14	93.3	
Otalgia					
Yes	—	—	2	13.3	0.487
No	12	100.0	13	86.7	
Tinnitus					
Yes	1	8.3	—	—	0.444
No	11	91.7	15	100.0	
Deafness					
Yes	1	8.3	—	—	0.444
No	11	91.7	15	100.0	

**Table 4:** Sign resolution with povidone-iodine and clotrimazole.

Variables	Drug				*P* value
	Clotrimazole + lignocaine		Povidone iodine		
	*n*	%	*n*	%	
Tragal tenderness					
Yes	—	—	—	—	—
No	12	100.0	15	100.0	
Cartilaginous canal wall erythema					
Yes	2	16.7	—	—	0.188
No	10	83.3	15	100.0	
Cartilaginous canal wall edema					
Yes	1	8.3	—	—	0.444
No	11	91.7	15	100.0	
Bony canal wall erythema					
Yes	1	8.3	1	6.7	1.000
No	11	91.7	14	93.3	
Bony canal wall edema					
Yes	—	—	1	6.7	1.000
No	12	100.0	14	93.3	
Ear discharge in the canal					
Yes	—	—	—	—	—
No	12	100.0	15	100.0	
Tympanic membrane congestion					
Yes	—	—	2	13.3	0.487
No	12	100.0	13	86.7	
